# Domestic fire emergency escape plans among the aged in NSW, Australia: the impact of a fire safety home visit program

**DOI:** 10.1186/s12889-019-7227-x

**Published:** 2019-07-04

**Authors:** W. Kathy Tannous, Kingsley Agho

**Affiliations:** 1 School of Business & Translational Health Research Institute, Western Sydney University, Penrith, New South Wales 2751 Australia; 2 School of Science and Health, Western Sydney University, Penrith, New South Wales 2751 Australia; 3 Digital Health Cooperative Research Centre, The Rocks, New South Wales, 2000 Australia

**Keywords:** Home fire, Escape plan, Fire safety, High-risk individual, New South Wales

## Abstract

**Background:**

Domestic fire-related injuries and deaths among the aged remain a concern of many countries including Australia. This study aimed to assess the impact of a home fire safety visit project on domestic fire emergency escape plans among the 373 aged persons using multivariate analyses.

**Method:**

The study used data from a collaborative intervention program by three emergency agencies in New South Wales. It covered 373 older people at registration and 156 at post home visit follow-up. The five fire emergency escape plan outcome measures (participants having a working smoke alarm, finding out what to do if there was a fire at their home, making a plan to escape their home in the event of a fire, finding out how to escape their home in an emergency and finding out how to maintain their installed smoke alarm) were examined by adjusting for key characteristics of participants, using a generalized estimating equation (GEE) model that adjusted for repeated measures in order to examine the association between the home visit program and fire emergency escape plans.

**Results:**

There were significant improvements in participants’ likelihood of finding out what to do if there was a fire in their home [AOR; 95% CI 1.89 (1.59–2.26)], making a plan to escape their home [AOR; 95% CI 1.80 (1.50–2.17)], how to escape their home in an emergency [AOR; 95% CI 1.33 (1.07–1.66)] and how to maintain their smoke alarm [AOR; 95% CI 1.77 (1.48–2.12)]. Female participants were less likely to have a plan to escape their home in the event of a fire [AOR; 95% CI 0.86 (0.75–0.99)] and to find out how to escape their home in an emergency [AOR; 95% CI 0.71 (0.61–0.82)] compared with their male counterparts. Additionally, participants who spoke languages other than English at home were significantly less likely to have a working smoke alarm [AOR; 95% CI 0.88 (0.38–0.69)].

**Conclusion:**

Our findings suggest that home visit programs are able to increase fire safety of vulnerable and isolated older people.

## Background

The majority of fire-related injuries and deaths in developed countries occur in the home [1–5]. In the United States, most fire-related deaths among young, middle-aged and older people are caused by residential fires [6–8]. In 2012–13, more than three-quarters of all fire-related deaths in the United Kingdom occurred in residential dwellings [9, 10] . Similarly, more than half of all fire-related deaths in Australia in 2013 resulted from fires in residential dwellings [11, 12].

Globally, the very young and the very old are most vulnerable to residential fires [13–15] and, although the incidence of residential fire-related injuries is lower among the elderly population, the likelihood of older people dying from fire-related injuries is higher than for other age groups [16, 17]. Research also shows that, among the elderly, the risk of death in a residential fire becomes higher as age increases. For instance, in the United States, the likelihood of people having a fire-related death is 20% above the national average for those aged 65 years, rising to double the national average at 75 years and four times the national average at age 85 years [18, 19]. Similarly, in the United Kingdom from 1996 to 2000, the fire-related death rate among people aged 60–79 years was five times higher than for people aged 20–39 years, and for people aged 80 years or above it was ten times higher [20]. In Australia, from July 1996 to June 2004, persons aged 65 years or over were the main victims of residential fire-related fatalities [21].

In New South Wales (NSW), Australia, a study was undertaken into household preparedness for a storm disaster and how to access information to better prepare for such a disaster [22]. In addition, several Australian studies have focused on home fire safety. A study conducted by Bruck (2001), combined several strands of research - auditory sleep arousal thresholds, responsiveness to auditory signals during sleep and responsiveness to smoke detector alarms during sleep – with data on fire fatalities and characteristics of victims. However, limited research has been undertaken in NSW into domestic fire emergency escape plans for residential fire-related disasters [23]. Therefore, the current study aimed to use data from a home visit program to assess the relationships between a range of demographic factors and domestic fire emergency escape plans.

## Method

### Study sample

The study involved people who were participants in a state-based community resilience program called the Home Fire Resilience Project (HFRP). This program focused on emergency response agencies working in partnership to visit vulnerable persons requiring assistance with their smoke alarms and home fire safety, to install long lasting smoke alarms with battery life of ten years and to provide information on emergency preparedness.

The target population for the study was at-risk seniors who access Australian Red Cross’s (ARC) Telecross program, an initiative that uses trained volunteers to make daily phone calls to check on the wellbeing and safety of vulnerable and isolated clients. Telecross has 7600 clients across Australia, most of whom are aged 80 years and above, are predominantly female and live by themselves (Australian Red Cross 2016). The initial target group was the 1713 Telecross clients living in NSW. Most were contacted and 488 expressed an interest in participating in the program. The possible reasons for nonparticipation by all clients were the expression of interest requirements, of completion and posting a four-page registration form, and/or the home visit component of program. Of the 488 that were interested, 373 completed the registration process and, by 30 April 2016, 253 had received a home visit, four had been contacted by phone and 73 were waiting for a visit. Of those who received a home visit, 156 participated in the post-visit survey. The reasons for participants who completed the registration process drop-out at stage of the process included the regional focus of the home-visits in four areas (Northern Rivers, Hunter Valley, Central Sydney and Greater Sydney) where participants were most concentrated (85% of clients who expressed an interest in the project were located in these four regions), change of residence, inability of ARC volunteers to contact the client again and death of the client.

The key aims of the program were to help clients build resilience and strengthen their preparedness for home fire emergencies. The program was organized by NSW fire emergency services Fire & Rescue New South Wales (FRNSW) and New South Wales Rural Fire Services (NSWRFS) in partnership with the Australian Red Cross (ARC). FRNSW is the agency responsible for fires and rescues in cities and towns and NSWRFS is responsible for rural fire districts and bush fires. For the fire emergency services, HFRP was an extension of the Smoke Alarm and Battery Replacement (SABRE) program the NSW fire brigade had introduced in 2002. SABRE is delivered by firefighters in response to requests by local councils, community-based care organizations or individuals to have battery-operated photoelectric smoke alarms installed or previously installed alarms checked [24]. ARC proactively offered the fire safety and emergency preparedness program to its Telecross clients.

### Sample size

**Fig. 1:**
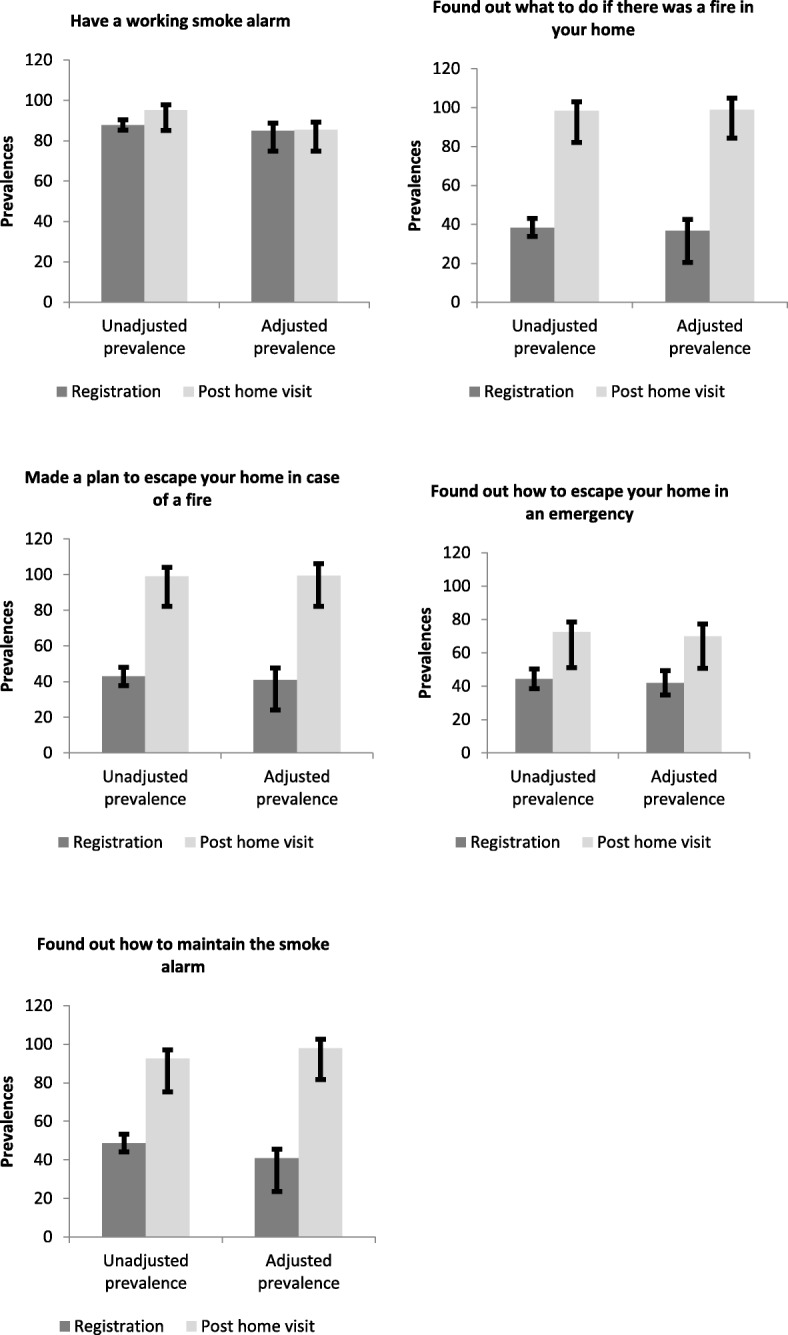
Prevalence rates: Unadjusted and adjusted for five fire emergency escape plan outcomes

The required sample size for this study was determined using a single population proportion formula. Past research has indicated that about 48% of elderly, vulnerable people are not adequately prepared to handle emergencies [25]. This study assumed a proportion of 50% of the population, and then based the calculation of total sample size on an error margin of 1%, at 90% power and 5% significance level for a two-sided test (Fig. 1). This gave a sample size of 260. Assuming a dropout rate of 42%, also based on earlier research (Tannous & Tetteh, 2016), a total sample size of approximately 370 participants was required. This sample size is sufficient to detect any statistical differences in an analysis of the home visit program and emergency preparedness among vulnerable older people.

### Data collection

Sociodemographic data on the participants were available at the commencement of the program, having been collected and put into the ARC database by Telecross volunteers when the clients initially accessed the Telecross service. These data included gender (about three quarters were female), age (average age was 81 years, with individual ages ranging from 48 to 97 years), country of birth (40% were born in Australia including five clients who identified as being indigenous), housing status (12% were social housing tenants) and language spoken at home (97% spoke English as their main language).

Subsequent data were collected by Telecross volunteers during the home visits, which commenced in July 2015, and the follow-up survey that started in early 2016. The follow-up period was not standardized as it was dependent on sourcing, training and availability of volunteers. The period of visits by the volunteers from the date of participants’ registration ranged from 1 month to 8 months. The volunteers were located in the same areas as the clients. At the start of program implementation, all volunteers were offered training in data collection and recording methods and 45 ARC volunteers undertook the training. Subject specialists delivered one-day training sessions in each of the four target regions. Additional 11 volunteers who joined the program after completion of the formal training sessions received one-on-one mentoring from previously-trained volunteers. They were recruited as the ratio to volunteers to clients was changed early in the program from one volunteer to a client to two volunteers for safety reasons.

The aims of the initial home visits were to engage the clients in conversations that prompted them to think about the steps they could take to prepare themselves better for an emergency incident and to provide them with a client preparedness kit consisting of information booklets, fact sheets, smoke alarms, batteries, magnets and wooden spatulas with printed messages. The follow-up survey was aimed to obtain information that would enable the program sponsors to assess the effectiveness of the interventions in improving client resilience and preparedness.

The United Nations Office for Disaster Reduction (UNISDOR) defines preparedness as the knowledge and capacities developed by individuals to effectively anticipate the likely impact of hazardous events or conditions [26]. In the first half of 2015, experienced ARC staff formulated an overarching question and five sub-questions to assess emergency preparedness that would be suitable for this project. Telecross volunteers presented these questions unchanged to clients at two different times: at registration for the program and after delivery of the home visit or telephone call by the ARC volunteers. The overarching question was: “What have you done to be safe from a fire in your home?” The five sub-questions were: “Have you found out what to do if there was a fire in your home?”; “Have you made a plan to escape your home in case of a fire?”; “Have you found out how to escape your home in an emergency?”; “Do you have a working smoke alarm?”; and “Have you found out how to maintain the smoke alarm?” On each occasion, program participants were provided with three possible responses: “Yes”, “No” and “Unsure”.

ARC provided the data collected by the Telecross volunteers to the research team responsible for project evaluation. Data were provided in de-identified form to the authors and ethics approval was obtained from Western Sydney University Human Research Ethics Committee (H11159). The research team coded and cleaned the data for analysis and comprised of ARC administrative data, volunteers entered information and response agency information. It then tested for associations between the data on socio-demographic factors and the responses to the different questions on fire safety in the home.

### Dependent variables

The dependent variables related to fire emergency preparedness and escape plans: (1) participants having a working smoke alarm; (2) participants finding out what to do if there was a fire at their home; (3) participants making a plan to escape their home in the event of a fire; (4) participants finding out how to escape their home in an emergency; and (5) participants finding out how to maintain their installed smoke alarms.

### Potential confounders

Our choice of potential confounding factors (the socio-demographic variables) was based on previous literature on various factors relating to home fire emergencies. These potential confounders included (a) age of a resident [16, 17, 27]; (b) country of birth [17, 28, 29]; (c) gender of the participants [17, 27]; (d) type of living conditions [28, 29]; (e) language other than English spoken at home [30]; and (f) ethnicity [28, 29].

There are several other confounding factors that may have affected the findings of our study. First, the time lag between the program delivery visit and the follow-up survey visit ranged from a few weeks to several months. Since increasing forgetfulness is a common characteristic of the ageing, data collected soon after the program delivery visit may be more reliable than that collected later. Second, the methods of collecting data were different for the two home visits. For the program delivery visit, clients answered the questions on paper, either independently or with the support of a volunteer. For the follow-up survey visit, answers were provided orally to volunteers, either face-to-face during the follow-up survey visit or over the telephone for four clients who did not receive a face-to-face visit.

### Statistical analyses

Descriptive analyses were carried out on information from the 373 participants gathered at registration and from the 156 participants in the follow-up survey. The preliminary analyses involved frequency tabulations and summary statistics of all collected characteristics variables.

Outcome measures for a given participant are repeated measures over visit time (at registration and during the follow-up survey). Hence, a generalized estimating equations (GEE) model that adjusts for repeated measures was used. For binary outcomes a logit link with a binomial distribution for the outcome was used. The GEE estimates were translated into an odds ratio. A multivariate GEE was used to adjust for potential confounders. The GEE estimates were translated into odds ratios using STATA® version 14.1 software (2015, Stata Corporation, College Station, TX, US). All tests were two-sided, and *p*-values < 0.05 were considered statistically significant.

## Results

### Baseline characteristics

Table 1 shows some characteristics of the 373 participants at registration. The majority were female (~ 76%), and more than 50% were born outside Australia. A high proportion of the participants were non-Aboriginal (~ 99%). Most of the participants lived in private housing (~ 88%) and spoke English at home (~ 97%). The mean age of the participants was 81 years, with individual ages ranging from 48 to 97 years.

**Table 1:** Characteristics of participants at registration (*n* = 370)

Variables	*n*	%
Gender		
Male	49	24.5
Female	151	75.5
Age mean (sd)	82.3 (9.0)	
Country Of Birth		
Australia	153	41.4
Others	217	58.7
*Aboriginality*		
*Aboriginal*	5	1.4
*Others*	365	98.7
*Living conditions*		
*Private*	191	95.5
*Public*	9	4.5
*Language spoken at home*		
*English*	360	97.3
*Others*	10	2.7

### Univariate analysis

Table 2 summarizes the unadjusted odds ratios of the associations between some of the demographic characteristics of the participants and the various outcome measures of domestic fire escape plans. The odds of a participant finding out what to do in the event of a domestic fire [odds ratio (OR): 1.82, 95% confidence interval (CI): 1.53, 2.17), *p*-value (p) < 0.001], making a plan to escape the home in the event of a fire [OR: 1.77, 95% CI: (1.47, 2.13), *p* < 0.001)], finding out how to escape the home in an emergency [OR: 1.32, 95% CI: (1.05, 1.66), p < 0.001] and finding out how to maintain a smoke alarm [OR: 1.55, 95% CI: (1.29, 1.87), p < 0.001] were significantly higher among respondents after the home visit compared to those of respondents at the time of registration.

**Table 2:** Unadjusted Odd ratios for those who have a working smoke alarm, found out what to do if there was a fire in your home, made a plan to escape your home in case of a fire found out how to escape your home in an emergency and found out how to maintain the smoke alarm

Variables	Have a working smoke alarm			Found out what to do if there was a fire in your home			Made a plan to escape your home in case of a fire			Found out how to escape your home in an emergency			Found out how to maintain the smoke alarm		
	OR	(95%CI)	*P* value	OR	(95%CI)	*P* value	OR	(95%CI)	*P* value	OR	(95%CI)	*P* value	OR	(95%CI)	*P* value
Visits															
Registration	1.00			1.00			1.00			1.00			1.00		
Post home visit	1.08	(0.96,1.20)	0.199	1.82	(1.53, 2.17)	< 0.001	1.77	(1.47, 2.13)	< 0.001	1.32	(1.05,1.66)	0.016	1.55	(1.29,1.87)	< 0.001
Age in years	1.00	(0.99, 1.01)	0.580	1.00	(0.99, 1.01)	0.778	1.00	(0.99, 1.01)	0.544	1.00	(0.99, 1.01)	0.412	1.00	(0.99, 1.01)	0.431
Country Of Birth															
Australia	1.00			1.00			1.00			1.00			1.00		
Others	0.97	(0.91, 1.03)	0.369	0.94	(0.85, 1.04)	0.255	0.95	(0.86, 1.06)	0.342	1.02	(0.91, 1.14)	0.695	1.06	(0.95, 1.17)	0.255
Gender															
Male	1.00			1.00			1.00			1.00			1.00		
Female	0.97	(0.90, 1.04)	0.431	0.88	(0.78,0.99)	0.037	0.88	(0.78, 0.99)	0.037	0.80	(0.71, 0.90)	< 0.001	0.91	(0.81, 1.02)	0.102
Living conditions															
Private	1.00			1.00			1.00			1.00			1.00		
Public	0.91	(0.75, 1.11)	0.354	0.97	(0.70, 1.35)	0.863	0.88	(0.62, 1.26)	0.486	0.95	(0.67, 1.35)	0.780	0.84	(0.60, 1.17)	0.291
Language spoken at home															
English	1.00			1.00			1.00			1.00			1.00		
others	0.82	(0.67, 1.00)	0.050	0.99	(0.71, 1.37)	0.948	0.85	(0.61, 1.18)	0.328	1.06	(0.75, 1.51S)	0.731	0.86	(0.61, 1.22)	0.399

Females had lower odds of finding out what to do in the event of a domestic fire [OR: 0.88, 95% CI: (0.78, 0.99), *p* = 0.037)], making a plan to escape the home in the event of a fire [OR: 0.88, 95% CI: (0.78, 0.99), p = 0.037)] and finding out how to escape the home in an emergency [OR: 0.80, 95% CI: (0.71, 0.90), *p* < 0.001)] compared to males.

The odds of having a working smoke alarm were significantly higher among respondents whose main language at home was English compared to those whose main language at home was not English [OR: 0.80, 95% CI: (0.71, 0.90), *p* < 0.001)].

### Multivariate analysis

Table 3 shows the associations between the demographic characteristics of the respondents and the different outcome measures of domestic fire emergencies when other variables are controlled for. The odds of a participant finding out what to do in the event of a domestic fire [OR: 1.89, 95% CI: 1.59, 2.26), *p* < 0.001], making a plan to escape the home in the event of a fire [OR: 1.80, 95% CI: (1.50, 2.17), p < 0.001)], finding out how to escape the home in an emergency [OR: 1.33, 95% CI: (1.07, 1.66), p < 0.001] and finding out how to maintain a smoke alarm [OR: 1.77, 95% CI: (1.48, 2.12), *p* < 0.001] were significantly higher among respondents after the home visit than at the time of registration.

**Table 3:** Adjusted Odd ratios for those who have a working smoke alarm, found out what to do if there was a fire in your home, made a plan to escape your home in case of a fire found out how to escape your home in an emergency and found out how to maintain the smoke alarm

Variables	Have a working smoke alarm			Found out what to do if there was a fire in your home			Made a plan to escape your home in case of a fire			Found out how to escape your home in an emergency			Found out how to maintain the smoke alarm		
	AOR	(95%CI)	*P *value	AOR	(95%CI)	*P* value	AOR	(95%CI)	*P* value	AOR	(95%CI)	*P *value	AOR	(95%CI)	*P* value
Visits															
Registration	1.00			1.00			1.00			1.00			1.00		
Post home visit	1.07	(0.96,1.20)	0.232	1.89	(1.59, 2.26)	< 0.001	1.80	(1.50, 2.17)	< 0.001	1.33	(1.07,1.66)	0.01	1.77	(1.48,2.12)	< 0.001
Age in years	1.00	(0.99, 1.01)	0.574	1.00	(0.99, 1.01)	0.74	1.00	(0.99, 1.01)	0.89	1.00	(0.99, 1.01)	0.38	1.00	(0.99, 1.01)	0.54
Country Of Birth															
Australia	1.00			1.00			1.00			1.00			1.00		
Others	1.06	(0.96, 1.16)	0.235	0.93	(0.81, 1.08)	0.36	0.97	(0.83, 1.13)	0.66	1.05	(0.90, 1.24)	0.53	1.06	(0.92, 1.22)	0.44
Gender															
Male	1.00			1.00			1.00			1.00			1.00		
Female	0.98	(0.90, 1.08)	0.744	0.92	(0.78,1.06)	0.24	0.86	(0.75, 0.99)	0.05	0.71	(0.61, 0.82)	< 0.001	0.89	(0.77, 1.03)	0.13
Living conditions															
Private	1.00			1.00			1.00			1.00			1.00		
Public	0.94	(0.78, 1.13)	0.526	1.06	(0.78, 1.43)	0.72	1.03	(0.74, 1.43)	0.85	1.05	(0.75, 1.47)	0.99	0.91	(0.66, 1.24)	0.53
Language spoken at home															
English	1.00			1.00			1.00			1.00			1.00		
others	0.88	(0.38, 0.69)	< 0.001	0.76	(0.49, 1.19)	0.23	0.72	(0.50, 1.15)	0.16	0.67	(0.41, 1.09)	0.10	0.70	(0.44, 1.10)	0.12

Females had lower odds of making a plan to escape the home in the event of a fire [OR: 0.86, 95% CI: (0.75, 0.99), *p* = 0.050)] and finding out how to escape the home in an emergency [OR: 0.71, 95% CI: (0.61, 0.82), p < 0.001)] compared to males.

### Unadjusted and adjusted prevalence of the dependent variables

As shown in Fig. 1, the unadjusted prevalence of having a working smoke alarm was slightly higher post home visit compared to during registration, and the adjusted prevalence was almost the same for both post home visit and registration, although these were not statistically significant, due to an overlap of the error bars. For both unadjusted and adjusted measures, the prevalence of finding out what to do in the event of a home fire was significantly higher at post home visit than at registration. The unadjusted and adjusted prevalence of making a plan to escape a home fire was significantly higher at post home visit compared to registration. For both unadjusted and adjusted measures, the prevalence of finding out how to escape from a home in an emergency was higher at post home visit compared to registration. Furthermore, the unadjusted and adjusted prevalence of finding out how to maintain a smoke alarm was significantly higher at post home visit compared to registration.

## Discussion

In this study, we explored the impact of a fire safety home visit program on domestic fire escape plans among the elderly in the state of NSW, Australia. We also investigated the relationships among demographic characteristics of participants and the various outcome measures of escape plans in the event of a domestic fire. We found that, overall, the odds of participants having successfully developed an escape plan were higher after the home visit compared to during the registration period of the program.

Research in the United States [31, 32] has shown that having a fire escape plan constitutes only one component of the fire safety readiness of a household, and may not be sufficient to ensure protection from fire-related deaths or injuries. According to that study, to ensure that members of households understand the plan, are able to implement it and can justify it based on the conditions of the home and the needs of the residents, practicing the plan is essential [33].

The essence of the HFRP home visit program was to showcase various escape plans and to remind participants of the significance of practising their own escape plans. The home visit program seems to have been successful, as the odds of having a successful escape plan were higher after the program home visit than at the time of registration. This could be due in part to the tendency of elderly persons to forget what they have been taught and therefore to have benefited from the reminders provided by the program.

In the United States, one study [34] found that households with children were more likely to have a house fire and also to practice a fire escape plan. According to that study, nearly three-quarters of households with children whose ages ranged from five to 17 years reported having a fire escape plan, and about 85% of households with children aged five years or younger reported having discussed or practiced a fire escape plan. The study [34] also found that households with older people were less likely to have or practice a fire escape plan. The elderly are an important prevention focus, like lack of mobility due to old age is an important handicap when trying to escape a house fire [35]. As people age, their cognitive and sensory functions decline naturally and their mobility decreases, making older adults less able to recognize fire hazards and perhaps more likely to inadvertently engage in riskier behaviors [36, 37]. This was one important justification for the home visit program targeting the aged.

Our study revealed that males were significantly more likely than females to find out what to do in the event of a home fire, make a plan to escape the home in the event of a fire and find out how to escape the home in an emergency. This is consistent with a previous study [38], which revealed that males exhibited better wayfinding skills than females in a home fire emergency.

Several researchers have found that the risk of becoming a fatality in a residential fire is higher when smoke alarms are not installed [39] and that this risk can therefore be substantially reduced by installing and maintaining a smoke alarm [40]. Many countries have introduced legislation making smoke alarm installation in residential dwellings mandatory. Regulations differ from country to country with regard to who is responsible for installation of the alarms; for instance, in rented dwellings either the owner or occupier may be responsible for ensuring the home is equipped with a functioning alarm.

In Australia, it is a regulatory requirement to have an installed smoke alarm in the home. In addition, to remove or in any way tamper with the smoke alarm is a felony with the exception being that the device is being maintained or replaced [41, 42]. The requirement for having an operational smoke alarm is detailed in the Building Code of Australia and applies to the different forms of residential accommodation including houses, units in apartment buildings, caravans, and boarding houses [43]. The responsibility of fitting and maintaining a smoke alarm in the suitable locations falls on the owner of the property. The type of smoke alarm that may be installed is the choice of the homeowner with the proviso that the specifications align with Australian Standard AS 3786. Most homeowners install battery powered smoke alarms, 1 year removal or non-removable 10-year, as they are able to fit them and do not require the services of a professional electrician [43]. For some properties, hard-wired powered alarms are mounted and may be interconnected but they are generally more expensive and require electrician service for installation and maintenance. Person with cognitive or physical challenges, such as the elderly, are able to have their batteries checked on their smoke alarms or have them installed by contacting their local fire brigade or rural fire service. However, the requirements this arrangement is that the individuals must supply the batteries and/or smoke alarm for the response personnel to undertake this activity. In terms of placement or location of the smoke alarm, it is advised that they be fitted in areas close to or around the entrance to bedrooms. For homes with multiple levels, it is a minimum requirement to have a smoke alarm in every level and advisable to have one in every bedroom and living space, including the garage [43]. A common misconception held by homeowners is to place the smoke alarm where there may be smoke, such as the kitchen or outside the bathroom. This results in the smoke alarms being regularly triggered by cooking smoke or steam from showers or hairdryers in the bathrooms. The usual action is for the battery to be removed or disassembled rendering the alarm non-operational. A more appropriate practice is to move the device to another area in the house or room to ensure that it does not provide a false negative triggers.

In our study we found that older people who spoke mainly English at home had significantly higher odds of having a working smoke alarm installed in their home. This finding is consistent with a previous study which showed that certain population sub-groups are less likely to own smoke alarms; in particular, households with lower incomes [44]. This is perhaps because poorer households may not be able to afford a smoke alarm; it might also explain why the unemployed are less likely to install alarms or replace flat alarm batteries. Those who do not speak English at home might be recent migrants who are more likely to live in poorer households, be unemployed, or be unaware of the regulatory requirements.

The process of delivering the program included a component on creation of a fire escape plan for the residents that had not had a plan already (or did not remember) and practice of the plan. This was conducted by the ARC volunteers and by the fire fighters or rural fire service. The second visit and/or survey was conducted following the provision of client preparedness kit, consisting of information material, smoke alarms, batteries, magnets and printed messages, and visit by the fire brigade. In the process of checking or preparing the residents’ home escape plan, there was the need to prepare a list of people to call and have a conversation with about their plan. This saw the proportion of residents having had a conversation about their escape plan at registration to follow-up rising from 27 to 58%. The persons they had conversations with about their plan were predominantly family, friends and neighbors. At the end of the program, more than 60% of the residents stated that in case of an emergency, they were aware of who can help them in their neighborhood and two-thirds had swapped telephone numbers with their neighbors. The unintended benefit of the program was the increased social connectedness of residents.

Our study had a recognized limitation: data on the presence and practice of fire escape plans were based on self-reports from participants. The variables may be subject to social desirability bias and thus some measures may have been overstated. However, there are few other options for measuring the variables used in this analysis. The participants’ responses were validated by ARC volunteers’ observations of their homes and by their informal conversations.

Despite this limitation, the study has important implications for policy. Since the cognitive and sensory functions of the aged decline naturally over time, they tend to forget more easily. This may explain their inability to maintain their smoke alarms or remember their escape plans. Consequently, these older people can benefit from timely reminders, possibly by telephone calls or text messages, to rehearse their escape plans and change the batteries of their smoke alarms when necessary.

The benefits of the program for older, vulnerable and isolated people was the increased fire safety in their home coupled. This is coupled with greater knowledge of their health and frailty conditions by the emergency response agencies. The program costs are predominantly the client preparedness kit and the volunteer training material. These have already been incurred and much of the ongoing work is undertaken by volunteers suggesting that the continued program delivery would be at low marginal cost and should be continued. The follow-up program could be offered first to all Telecross clients who did not participate in the initial program, since ARC already has their details in its database and Telecross volunteers are in daily phone contact with them. However, some changes could be considered in the initial program. First, registration could be made simpler and more client-friendly. Rather than requiring prospective clients to fill out an expression of interest form that had to be posted to ARC, necessary registration details could be collected during the daily Telecross phone calls to clients. Second, because Telecross trainers and volunteers are geographically dispersed, volunteers are continually joining and leaving the program and face-to-face delivery is expensive and difficult to organise, training for volunteers may be available in online format. Third, many of the Telecross clients have severe mobility and/or income constraints and cannot easily acquire a smoke alarm and batteries. An important component of the program is the free provision of smoke alarms and batteries for all participants and this aspect needs to be maintained. The SABRE program that is available to persons aged 65 and over requires them to source the smoke alarms and their batteries and the brigade would install them. Fourth, visits by Telecross volunteers, who focus on emergency preparedness, should be separate from visits by the fire emergency agencies, which are used to undertake installation of the smoke alarm and home fire safety activities. If joint visits are undertaken, training may be needed to ensure friendly and effective cooperation between the ARC volunteers and the fire emergency agency staff, perhaps by the inclusion of modules on the different roles and challenges faced by the ARC and the fire emergency agencies. This conclusion stemmed from feedback that was provided by volunteers on the challenges encountered in coordinating the different calendars of 2 volunteers and 2 response agency personnel for the visit. As well, it was also identified that having four persons in the client’s home, in most cases older frail women living on their own, was overwhelming. Finally, to facilitate monitoring and control of the program, data coding, collection and recording need to be organised and standardised to facilitate clear presentation and easy analysis of results.

## Conclusion

The HFRP home visit program achieved its intended aim of increasing fire safety of vulnerable older people in NSW. In addition to ensuring that participants have a working smoke alarm with battery life of ten years, the information provided by ARC volunteers also offered a deeper awareness of home fire emergency preparedness and community connectedness. The HFRP program resources entailed the development of client preparedness kit. The other aspects of FRNSW or RFS visits are part of those organization’s community safety practice. The ARC facilitated the reach to a most vulnerable and isolated group. The unplanned benefit of the program was the increased social connectedness of residents with family, friends and neighbors. At the end of the program, more than two-third of the residents stated that in case of an emergency, they were aware of who can help them in their neighborhood and had swapped telephone numbers with their neighbors.

## Data Availability

Data can be made available upon request from the corresponding author in aggregate form only.

## Abbreviations

AOR: Adjusted odds ratio
ARC: Australian Red Cross
CI: Confidence interval
FRNSW: Fire & Rescue New South Wales
GEE: Generalised estimating equations
HFRP: Home Fire Resilience Project
HFSC: Home Fire Safety Checks Program
NSW: New South Wales
NSWRFS: New South Wales Rural Fire Service
SABRE: Smoke Alarm and Battery Replacement
UNISDOR: United Nations Office for Disaster Reduction

## References

[1] Mock C, Peck M, Juillard C, Meddings D (2011). Burn Prevention: Success Stories, Lessons Learned.

[2] Mailstop F (2009). AFG prevention, and Saftey Grants, Global Concepts in Residential Fire Safety Part 3–Best Practices from Canada*,* Puerto Rico, Mexico, and Dominican Republic.

[3] Schaenman P (2007). Global concepts in residential fire safety part 1—best practices from England, Scotland, Sweden and Norway.

[4] Haynes HJ. Fire loss in the United States during 2014. National Fire Protection Association. Quincy: Fire Analysis and Research Division; 2015.

[5] Mailstop F (2008). Global concepts in residential fire safety: part 2 – best practices from Australia, New Zealand and Japan.

[6] McGwin G, Chapman V, Curtis J, Rousculp M (1999). Fire fatalities in older people. J Am Geriatr Soc.

[7] Yau RK, Marshall SW (2014). Association between fire-safe cigarette legislation and residential fire deaths in the United States. Inj Epidemiol.

[8] Bishai D, Lee S (2010). Heightened risk of fire deaths among older African Americans and native Americans. Public Health Rep.

[9] Holborn P, Nolan P, Golt J (2003). An analysis of fatal unintentional dwelling fires investigated by London fire brigade between 1996 and 2000. Fire Saf J.

[10] Troitzsch JH (2016). Fires, statistics, ignition sources, and passive fire protection measures. J Fire Sci.

[11] Australian Bureau of Statistics. Australian Social Trends 2000, Catalogue No: 4102.0, Canberra, Australia, 2000.

[12] Productivity Commission, Report on Government Services. 2016, Productivity Commission.

[13] Istre GR, McCoy M, Carlin DK, McClain J (2002). Residential fire related deaths and injuries among children: fireplay, smoke alarms, and prevention. Inj Prev.

[14] Notake H, Sekizawa A, Kobayashi M, Mammoto A, Ebihara M (2004). How to save the lives of vulnerable people from residential fires. Human behaviour in fire: proceedings of the 3rd international symposium.

[15] Miller I. Human Behaviour Contributing to Unintentional Residential Fire Deaths, 1997-2003. Wellington: New Zealand Fire Service Commission; 2005.

[16] Zhang G (2006). Fire safety among the elderly in Western Australia. Fire Saf J.

[17] Istre GR, Lee AH, Lee HC, Clinton M (2001). Deaths and injuries from house fires. N Engl J Med.

[18] United States Fire Administration. Older adults and fire. Topical Research Series, 1 (5). 2001; Available from: https://www.hsdl.org/?view&did=19518. [cited 2016 August 7]

[19] McDonald EM, Mack K, Shields WC, Lee RP, Gielen AC (2018). Primary care opportunities to prevent unintentional home injuries a focus on children and older adults. Am J Lifestyle Med.

[20] Holborn PG. The real fire library: Analysis of fatal fires 1996-2000. London Fire Brigade - Fire Investigation Group, London Fire and Emergency Planning Authority, Nov. 2001.

[21] Australasian Fire Authorities Council, Accidental fire fatalities in residential structures: Who's at risk? 2005 (March 2005), Australasian Fire Authorities Council, Melbourne, Australia.

[22] Cretikos M, Eastwood K, Dalton C, Merritt T, Tuyl F, Winn L, Durrheim D (2008). Household disaster preparedness and information sources: rapid cluster survey after a storm in New South Wales, Australia. BMC Public Health.

[23] Tannous WK, Whybro M, Lewis C, Ollerenshaw M, Watson G, Broomhall S, Agho KE (2016). Using a cluster randomized controlled trial to determine the effects of intervention of battery and hardwired smoke alarms in New South Wales, Australia: home fire safety checks pilot program. J Saf Res.

[24] Fire & Rescue New South Wales, Annual Report 2014/15. 2015.

[25] Tannous WK, Tetteh VW (2016). Evaluation of vulnerable communities resilience project for Fire & Rescue new South Wales Final Report.

[26] United Nations Office for Disaster Risk Reduction, Terminology (2016). The United Nations Office for Disaster Risk Reduction.

[27] Zhang X, Li X, Hadjisophocleous G (2013). A probabilistic occupant evacuation model for fire emergencies using Monte Carlo methods. Fire Saf J.

[28] Edelman LS (2007). Social and economic factors associated with the risk of burn injury. Burns.

[29] Duncanson M, Woodward A, Reid P (2002). Socioeconomic deprivation and fatal unintentional domestic fire incidents in New Zealand 1993–1998. Fire Saf J.

[30] Shai Donna (2006). Income, Housing, and Fire Injuries: A Census Tract Analysis. Public Health Reports.

[31] Ahrens M. Home strucutre fires, 2018. National Fire Proetction Association, Quincy, Massachusetts, United States. https://www.nfpa.org/-/media/Files/News-and-Research/Fire-statistics-and-reports/Building-and-life-safety/oshomes.pdf.

[32] Centers for Disease Control and Prevention (2008). Preventing residential fire injuries, in Fact Sheet, Centers for Disease Control and Prevention.

[33] Thompson N, Waterman M, Sleet DA (2004). Using behavioral science to improve fire escape behaviors in response to a smoke alarm. J Burn Care Res.

[34] Yang J, Peek-Asa C, Allareddy V, Zwerling C, Lundell J (2006). Perceived risk of home fire and escape plans in rural households. Am J Prev Med.

[35] Runyan CW (2005). Risk and protective factors for fires, burns, and carbon monoxide poisoning in US households. Am J Prev Med.

[36] Federal Emergency Management Agency. Fire risk to older adults in 2010. Topical Fire Report Series 2014;14(9). Available from: http://https://www.usfa.fema.gov/downloads/pdf/statistics/v14i9.pdf.

[37] United States Fire Administration, Fire Risk in 2014, Tropical Report Series 17(7), 2016, Emmitsburg. Maryland: U.S. Department of Homeland Security, National Fire Data Center, United States; 2016.

[38] Tang C-H, Wu W-T, Lin C-Y (2009). Using virtual reality to determine how emergency signs facilitate way-finding. Appl Ergon.

[39] Ahrens M (2004). US experience with smoke alarms and other fire detection/alarm equipment.

[40] Marshall SW, Runyan CW, Bangdiwala SI, Linzer MA, Sacks JJ, Butts JD (1998). Fatal residential fires: who dies and who survives?. JAMA.

[41] Ahrens M, Smoke alarms in U.S. home fires, National Fire Protection Association, Quincy, Massachusetts, United States. January 2019. https://www.nfpa.org/-/media/Files/News-and-Research/Fire-statistics-and-reports/Detection-and-signaling/ossmokealarms.pdf.

[42] Senate Legal and Constitutional Affairs Committee (2016). Use of smoke alarms to prevent smoke and fire related deaths.

[43] New South Wales Government, Department of Planning, New smoke alarm requirements: Owners of houses, residential flats and units. Fact sheet 1. 2006; available from: https://www.planning.nsw.gov.au/-/media/Files/DPE/Other/smoke-alarms-new-smoke-alarm-requirements-owners-of-houses-residential-flats-and-units-2006-03.pdf.

[44] McKnight R, Struttmann T, Mays J (1995). Finding homes without smoke detectors: one step in planning burn prevention programs. J Burn Care Res.

